# Clinical Lectures1Read at the Sixth Annual Meeting of the Association of Veterinary Faculties and Examining Boards of North America, September 7, 1899.

**Published:** 1899-11

**Authors:** S. J. J. Harger

**Affiliations:** Professor of Anatomy and Zoötechnics, Veterinary Department, University of Pennsylvania


### CLINICAL LECTURES.[1]

BY S. J. J. HARGER, V.M.D.,

PROFESSOR OF ANATOMY AND ZOÖTECHNICS, VETERINARY DEPARTMENT, UNIVERSITY OF PENNSYLVANIA.

WHEN our Secretary assigned to me the subject "Clinical Lectures," I had no intimation whether or not there is any one special phase of the subject, in preference to any other, which was to be discussed, and hence I will confine myself to a few general remarks.

It is the custom among educational institutions, to which class veterinary schools belong, for the instructors to hold meetings or institutes, as they are called, to discuss and improve their methods of teaching, in order to make their instruction more effective and more simple of comprehension by the young mind.

It is very fortunate that this Association has successfully passed through the stormy scenes of the first few years of its existence in adjusting the curricula and the length of the scholastic course, and can now turn its attention to rejuvenating the methods of instruction which in veterinary schools have for years been almost empirical. While, for the future, the curriculum must be broadened to meet new economic and sanitary demands, yet as long as our needs or pleasures require domestic animals—and their day is not passed yet—animal diseases must be treated and their clinical study and instruction to students not neglected.

From time immemorial, and with pleasant recollections, every medical student has been familiar with the medical and the surgical clinic as a distinct part of his instruction, and has visited the various hospitals to acquaint himself with the methods of different clinicians. On the contrary, and without offering an uncalled-for critique, we may say that in veterinary schools formal clinical lectures with detailed remarks have received but little attention—at least not as much as they deserve. Likewise, considering the small number of students in veterinary schools, the old, trodden paths of the present didactic courses are susceptible of improve-

[1] Read at the Sixth Annual Meeting of the Association of Veterinary Faculties and Examining Boards of North America, September 7, 1899.

ment, even if it would be with an apparent detraction from the routine dignity of the professional incumbent.

Clinical instruction may be grouped under four headings:

1. Ordinary dispensary work.
2. Routine outdoor clinics.
3. Assignment of cases in the hospital.
4. Didactic clinical lectures.

All of these have their advantages, and, in order to obtain sufficient material, must be resorted to. The first and second groups may really be included under the second, the out-clinic, which furnishes to us the bulk of our clinical material. The observation of the course and the treatment of a sick animal in a hospital is of such advantage that it need not be dwelt upon, and the thorough performance of such duties should be insisted upon by the clinical staff and their assistants.

The advantage of clinical lectures deserves a few words as one of many parts that make a complete whole. Personally, I very much favor such lectures, and advocate their general introduction into the curriculum—literally signifying bedside instruction, instruction in the presence of the patient, there is no more forcible way than this of fixing upon the receptive mind facts and principles. It means the complete analysis of the phenomena, immediate and collateral, of a case; of its connection with others of allied types, and the summarizing of logical conclusions. Compared with the outdoor clinic, the latter, continuing daily for an hour or two, covers a large number of miscellaneous cases, but has the disadvantage of being less formal and accompanied by less concentration of the student's mind; also the limited time given to each case, and sometimes the difficulty of a diagnosis on the spur of the moment, may forbid us to enter into any detailed discussions otherwise of much importance.

A clinical lecture should be thorough, methodical, and accurate as well as practical. Unless the clinician has all the necessary facts at his finger ends he should have a previous preparation. I find that no matter how simple or how often repeated the subject, a short preparation, a previous concentration of thought upon the subject-matter, always facilitates a lecture. In fact, a haphazard, at-random lecture is not only uninteresting, but it also does not add to the high esteem in which students should hold their teachers—a relation between student and teacher which the latter, if he desires to be successful and do effective work, should always encourage; besides, young men frequently unconsciously imitate and follow the methods and tactics of those in authority over them—another fact which we as teachers should bear in mind. Given a patient, the clinician inquires into the history and etiology of the disease; he points out systematically the symptoms, their interpretation, and their connection with one or several organs, compares them with the symptoms of other diseases which, in at least some respects, might resemble the one before him, and thus makes a rational differential diagnosis and prescribes a rational treatment. This is the inductive method of teaching. From a few abstract phenomena he evolves a series of intelligible facts. Instructive demonstrations may here also be given by means of fresh or dried dissected specimens or mechanical contrivances. All this is accomplished while he holds the student's undivided attention.

The student not only receives the benefit of the particular facts thus imparted to him—teaching by fact, but he receives a higher form of instruction—teaching by principle. He becomes imbued with a methodical procedure, as, for instance, in a demonstration in anatomy, details are not overlooked, his perception becomes more acute, he learns how to analyze clinically a disease, sees it in all its relations with other and allied conditions, can draw rational conclusions, and is less susceptible of error.

Clinical lectures are the practical adjuncts to medicine, surgery, pathology, anatomy, and physiology—the object-lesson of the primary school. The courses in the more practical subjects, such as medicine and surgery, are usually somewhat crowded. The mind perceives only through the ear, and time will not allow of the reception of knowledge through the eye. Principles and facts are only repeated in words, and not by actual demonstration, and some of these must be actually omitted—for example, the movements and attitudes of the members in the various lamenesses and the reason for the same; surgical operations, with different procedures, according to circumstances; the use of dissected specimens to illustrate regional anatomy and the actions of the muscles; medical cases of a typical and obscure form; special modes of treatment, such as saline transfusion, etc. I believe that two hours weekly could be well appropriated to this purpose, and, whenever at hand, cases of special interest may be selected. Besides, a student would receive the benefits of the skill and experience of different clinicians and operations. This may, perhaps, seem like unnecessary repetition of instruction, but anyone who has had any experience with students knows its value.

In anticipation of a stated lecture on a subject, in order to be more effective, the instructor may in advance inquire into the cause, prevention, and lesion of a certain diseased condition, investigate the best mode of treatment, and make statistical collection. This is, therefore, an incentive to original investigation.

Some of the European hospitals, such as those of Vienna and Berlin, are renowned the world over for their clinics, which are visited by physicians from all parts. From the nature of the circumstances we cannot expect as much as this from veterinary clinics, although with strong efforts they could be made to be of interest to the general practitioner and strengthen his affiliations with veterinary science and veterinary schools.

With these few remarks, let me state that if I have not struck the keynote of this subject I hope some one in this Association will.

